# Prevalence and severity of atherosclerosis in extra cranial carotid arteries in Nigeria: an autopsy study

**DOI:** 10.1186/1471-2261-12-106

**Published:** 2012-11-16

**Authors:** Erete I Erete, Olabiyi G Ogun, Olulola O Oladapo, Effiong E U Akang

**Affiliations:** 1 Department of Anatomy, College of Medicine, University of Ibadan, P O Box 14259, Ibadan, Nigeria; 2 Department of Pathology, College of Medicine, University of Ibadan, Ibadan, Nigeria; 3 Division of Cardiovascular Medicine, Department of Medicine, College of Medicine, University of Ibadan, Ibadan, Nigeria

**Keywords:** Extra cranial atherosclerosis, Plaques, Nigerians, Autopsy, Cardiovascular risk factors

## Abstract

**Background:**

There has been a paucity of autopsy studies on atherosclerotic lesions in Nigerians, the last one conducted at our centre being more than four decades ago. There has also been considerable epidemiological transition. The objective of the study was to determine the frequency, severity, pattern and distribution of atherosclerotic lesions in extra cranial carotid arteries (ECCA) in Nigerians at autopsy.

**Methods:**

ECCA of 30 consecutive Nigerian patients undergoing autopsy at a University teaching hospital were examined using the American Heart Association (AHA) histological grading and classification of atherosclerosis.

**Results:**

Atherosclerotic lesions of ECCA were present in 73.3% of the subjects with the right and the left carotid bifurcations (28.3%) being the most frequently affected sites. Using the AHA classification of atherosclerosis, a total of 176(73.3%) lesions were found in the 240 histological sections of blood vessels examined. Of these, 22.5% were types I, 22.5% were types II, 15.4% were type V, and 7.5% were type III. The VII to type IX lesions were rare. When these atherosclerotic lesions were grouped into mild, moderate and severe, 52.5% were mild lesions (types I-III); 18.3% were moderate lesions (types IV and V); and 2.5% were severe lesions (types VI to IX). The severe lesions were most frequently observed in the left carotid bifurcation (50%) and they first appeared in the age group 45–49 years. Age, hypertension and diabetes mellitus were strong risk factors for atherosclerosis.

**Conclusions:**

Compared with four decades ago there has been an apparent increase in severity and extent of ECCA atherosclerosis especially after the age of 45 years in autopsies from our centre. This change in the amount of atherosclerosis over time is possibly due to the epidemiologic transition. This may worsen the rise in stoke incidence within this community and as such, great effort should be made to follow-up and manage CVD risk factors within the community.

## Background

Atherosclerosis is a chronic progressive disease of arteries which usually presents clinically as cardiovascular disease (CVD) events such as myocardial infarction and stroke. CVD is the leading cause of death in many parts of the world with stroke accounting for the highest morbidity and mortality in sub Saharan Africa [1]. Cardiovascular risk factors, particularly, hypertension and dyslipidaemia are prevalent in Nigerian stroke patients [2]. These risk factors are on the increase in the sub region due to epidemiological transition from communicable to non-communicable diseases [3-5]. Biological factors such as race, age, sex, height, waist circumference (WC) and abdominal wall thickness (AWT) influence the distribution and pattern of atherosclerotic lesions [6]. A high frequency of extra cranial carotid arteries (ECCA) atherosclerosis of the carotid bifurcation was found in white population [7]. There has been a paucity of autopsy studies on atherosclerotic lesions in Nigerians, the last one conducted being more than four decades ago [8]. This study was carried out to determine the frequency, severity, pattern and distribution of atherosclerotic lesions in ECCA in Nigerians at autopsy. We also assessed the relationship between atherosclerosis and anthropometric variables.

## Methods

This study is a descriptive, cross-sectional survey, carried out on bodies referred to the Department of Morbid Pathology for post-mortem, in the University College Hospital, Ibadan, Oyo State, Nigeria between July 2009 and June 2010. Thirty consecutive cases ≥ 20 years of age who were predominantly Yorubas were the subjects used for the study. The study protocol was approved by the institutional ethical review committee (University of Ibadan/University College Hospital Ethical Committee assigned number: UI/EC/08/0096). Written informed consent to participate in the study was obtained from the next of kin relatives of the subjects. The clinical records of the subjects were retrieved to obtain their anthropometric data. Information sought included, age, gender, clinical diagnosis, past medical history, presence of cardiovascular risk factors such as hypertension, diabetes mellitus, dyslipidaemia, cigarette smoking, and alcohol intake. The post-mortem pathological findings including height, WC, and maximal AWT were also recorded.

To harvest the ECCA, a midline incision was made from the chin to the sternum and the skin flaps were dissected up to the angles of the jaws thus exposing the carotid sheaths on either side. The skin, superficial fascia, muscles and deep fascia were flipped to expose the carotid sheath and excise the ECCA. The entire length and branches of the common carotid artery, the external carotid artery and the extra cranial part of the internal carotid artery on either sides of the neck were excised. Detailed gross anatomic examination of the arteries was carried out to look for narrowing, occlusion, and any other anomaly or pathological changes. The arteries were then opened longitudinally to expose their intimal surfaces which were examined systematically for the presence, site and extent of fatty streaks, atherosclerotic plaques, thrombi and emboli. Each pathological report systematically drew the detailed anatomy of the vessels, the site and extent of atherosclerotic plaques, stenosis and occlusion.

The vessels were fixed in 10% buffered formalin solution and the tissues were processed in Leica EM TP4C automatic tissue processor. Sections were prepared using the Leica RM 2125 rotary microtome and stained with haematoxylin and eosin, or Masson’s trichrome stains for microscopic histology.

Atherosclerotic lesions were microscopically classified according to the modified American Heart Association (AHA) classification [7,9] as follows: 0 = no foam cells; 1 = single isolated foam cells; 2 = multiple foam cells (> 2 layers); 3 = pools of extracellular lipid (few or no cholesterol crystals); 4 = extracellular lipid with cholesterol crystals; 5 = fibrosis; 6 = surface defect, plaque haemorrhage or thrombosis; 7 = >50% of plaque area calcified (with/without lipid core); 8 = hyalinised fibrous plaque (no lipid core); and 9 = total occlusion. A frequency table was used to determine the frequency, distribution and pattern of atherosclerotic lesions in the left and the right common carotid arteries, the left and the right common carotid bifurcations, the left and the right external carotid arteries, and extra cranial part of the left and the right internal carotid arteries.

Data was reported as mean ± standard deviation. The relationship between categorical variables such as age, sex, height, WC, maximal AWT and atherosclerotic lesions of the ECCA were assessed using Chi-square. A p value of < 0.05 was accepted as significant. Pearson’s correlation coefficient test was used to determine the correlation between these variables.

## Results

The anthropometric variables of the studied subjects are as shown in Table 1. The study population consisted of equal number of male and female subjects with their ages ranging from 20 to 72 years with a mean of 40.4 ± 15.8 years. The medical records of the subjects studied showed that 23 (76.7%) had no identifiable risk factors for atherosclerosis whilst 7(23.3%) subjects had at least one risk factor for atherosclerosis. Amongst the later, hypertension alone was found in 20%, and hypertension combined with diabetes mellitus in 3.3%. Twenty five (83.3%) of the 30 subjects had atherosclerotic lesions in their ECCA of whom 54.5% were males and 45.5% were females. This gender distribution was not statistically significant, p = 0.62. The mean age of the subjects who had atherosclerotic lesions (47.1 ± 14.9 years) was significantly greater than that of the subjects who did not have atherosclerotic lesions (27.2 ± 6.7 years), p = 0.03. The mean age of males with atherosclerotic lesions (47.3 ± 12.2 years) was not significantly greater than the mean age of females (39.2 ± 16.6 years), with atherosclerotic lesions, p = 0.18. All 7 subjects who had either diabetes or hypertension had carotid artery atherosclerotic lesions. Similarly, all the 5 subjects who did not have atherosclerotic lesions had no identifiable underlying risk factors for atherosclerosis. The anatomical distribution of atherosclerotic lesions in the 25(83.3%) subjects who had atherosclerosis showed that the right and the left carotid bifurcations (28.3%) were most frequently affected, followed by the right and the left internal carotid arteries (27.4%). The remaining vessels had approximately similar plaque distribution ranging from 21.7 to 22.7%.

**Table 1:** Clinical and anthropometric variables in 30 subjects

**Variables**	**Female (n = 15)**	**Male (n = 15)**	**Total (n = 30)**	***P value***
Age (years **± SD**)	36.9 **±** 16.5	43.9 **±** 13.0	40.4 **±** 15.0	0.30
Past history				
Hypertension	2(6.7%)	4(13.3%)	6(20%)	0.67
Diabetes	1(3.3%)	0(0%)	1(3.3%)	0.173
Dyslipidaemia	0	0	0	
Smoking	0	0	0	
Height (cm **± SD**)	159.1 **±** 8.3	169.7 **±** 4.6	164.4 **±** 8.5	<0.001*
WC (cm **± SD**)	50.3 **±** 20.1	50.6 **±** 14.1	50.4 **±** 17.1	0.96
AWT (cm **± SD**)	3.7 **±** 2.3	2.6 **±** 1.7	3.2 **±** 2.1	0.10

*WC* (Waist circumference); *AWT* (Abdominal wall thickness);*Statistically significant p value.

A total of 176(73.3%) lesions were found in the 240 histological sections of blood vessels examined in the 30 subjects. The AHA classification of these lesions is shown in Table 2. In the 240 histological sections, 54 (22.5%) of the atherosclerotic lesions were types I and 54 (22.5%) were types II, 37 (15.4%) were type V, and 18 (7.5%) were type III. The VII to type IX lesions were rare. When these atherosclerotic lesions were classified into mild, moderate and severe, 126 (52.5%) were mild lesions (types I-III); 44 (18.3%) were moderate lesions (types IV and V); 6(2.5%) were severe lesions (types VI to IX). Severe atherosclerotic lesions were most frequently observed in the left carotid bifurcation (50%), the right carotid bifurcation (16.7%), the left common carotid artery (16.7%), and the right common carotid artery (16.7%).

**Table 2:** Distribution of atherosclerotic lesion types in extra cranial carotid arteries using the AHA classification

**Artery type**		**AHA Atherosclerotic Lesion Types**								
	**Lesion absent**	**Lesion present**								
	**Mild**	**Moderate**				**Severe**				
	**0**	**I**	**II**	**III**	**IV**	**V**	**VI**	**VII**	**VIII**	**IX**
**TOTAL = 240**	**64**	**54**	**54**	**18**	**7**	**37**	**5**	**0**	**1**	**0**
**%**	26.7	22.5	22.5	7.5	2.9	15.4	2.1	0	0.4	0
**Right common carotid artery**	10	8	5	0	2	4	1	0	0	0
**Left common carotid artery**	10	7	7	0	0	5	0	0	1	0
**Right internal carotid artery**	6	7	7	4	1	5	0	0	0	0
**Left internal carotid artery**	6	7	8	4	1	4	0	0	0	0
**Right carotid bifurcation**	5	3	11	2	2	6	1	0	0	0
**Left carotid bifurcation**	5	2	9	5	1	5	3	0	0	0
**Right external carotid artery**	11	11	3	1	0	4	0	0	0	0
**Left external carotid artery**	11	9	4	2	0	4	0	0	0	0

It was observed that the severity of atherosclerotic lesions progressed with age as shown in Table 3. Mild atherosclerosis first appeared in the age group 20–24 years; moderate atherosclerosis first appeared in the age group 25–29 years; severe atherosclerosis first appeared in the age group 45–49 years. The most severe lesion occurred in the age group 60–64 years in a subject who had diabetes and hypertension.

**Table 3:** Frequency of distribution of atherosclerotic lesions by age

		**Lesion absent**			**Lesion present**							
**Age group**	**Age frequency**		**Mild**		**Moderate**			**Severe**				**Total number of lesions**
		**0**	**I**	**II**	**III**	**IV**	**V**	**VI**	**VII**	**VIII**	**IV**	
**20**–**24**	**3**	22	0	2	0	0	0	0	0	0	0	**2**
**25**–**29**	**6**	24	6	8	0	0	2	0	0	0	0	**16**
**30**–**34**	**5**	8	10	12	5	4	1	0	0	0	0	**32**
**35**–**39**	**4**	8	15	9	0	0	0	0	0	0	0	**24**
**40**–**44**	**2**	0	0	4	4	0	8	0	0	0	0	**16**
**45**–**49**	**3**	2	7	5	3	2	2	3	0	0	0	**22**
**50**–**54**	**1**	0	3	3	2	0	0	0	0	0	0	**8**
**55**–**59**	**1**	0	0	4	2	0	2	0	0	0	0	**8**
**60**–**64**	**4**	0	5	7	2	1	15	1	0	1	0	**32**
**65**–**69**	**1**	0	0	0	0	0	7	1	0	0	0	**8**
**70**–**74**	**1**	0	8	0	0	0	0	0	0	0	0	**8**
**TOTAL**	**30**	**64**	**54**	**54**	**18**	**7**	**37**	**5**	**0**	**1**	**0**	**176**

Table 4 shows the distribution of atherosclerotic lesions in the subjects with risk factors for atherosclerosis. All the seven subjects with risk factors for atherosclerosis had atherosclerotic lesions. The six subjects who had hypertension alone had 48(27.3%) of the overall atherosclerotic lesions. Twenty-seven (56.3%) of these lesions were mild to moderate, while 21(43.8%) were severe. One subject with combined hypertension and diabetes mellitus had 8(4.5%) of the atherosclerotic lesions, out of which six lesions (75%) were moderate and two lesions (25%) were severe.

**Table 4:** Frequency of lesions in subjects who had risk factors of atherosclerosis

**ATHEROSCLEROTIC LESION TYPES**											
**Risk Factors**	**Number of subjects with risk factor**	**Mild**			**Moderate**		**Severe**				**Total No. of lesions**
		**I**	**II**	**III**	**IV**	**V**	**VI**	**VII**	**VIII**	**IX**	
**Hypertension**	6	12	13	2	1	20	0	0	0	0	**48**
**HTN & DM**	1	0	0	0	0	6	1	0	1	0	**8**
**Total**	**7**	**12**	**13**	**2**	**1**	**26**	**1**	**0**	**1**	**0**	**56**

*HTN & DM*: hypertension and diabetes mellitus.

Age and WC showed a positive correlation with carotid atherosclerosis (r = 0.60; p < 0.001). There was no correlation between lesions and height (r = 0.25; p > 0.05) and with AWT (r = 0.19; p > 0.05).

## Discussion

Our study is the first to use the AHA grading system of atherosclerosis which took into cognisance the extent and severity of the lesions in Nigerians. The findings from our descriptive study showed that the prevalence of atherosclerosis of ECCA in the subjects was 73.3% with 20.8% having advance lesions. Of the later, 18.3% had moderate and 2.5% had severe atherosclerosis. The commonest site of atherosclerosis was the right and left carotid bifurcations (28.5%) followed by the right and left internal carotid arteries (27.4%). The commonest risk factor was hypertension found in 20% of the subjects, and combination of hypertension and diabetes in 3.3%. We also found age to be a strong risk factor for atherosclerosis. The absence of dyslipidaemia and smoking in our study population may be attributed to the small sample size and the fact that this is a retrospective study that has its own inherent limitation.

Our study is the first to show that advance atherosclerosis occurs frequently at the rate of 20.8% in the ECCA of Nigerians. A relatively lower frequency and severity of atherosclerosis of the intracranial cerebral arteries was observed and documented as 13% in a study undertaken close to four decades ago in the same hospital [8]. This was a comparative autopsy study of cerebral atherosclerosis of Nigerians and Minnesota Caucasians in which the former had less severity and extent of atherosclerosis than the later. It was concluded then that the relatively short duration of hypertension in the Nigerian before death might be an important factor which did not permit progressive development of cerebral atherosclerosis. Although the risk factors, hypertension and diabetes mellitus in this study and ours remain the same, we observed an increased frequency, almost a doubling of the rate of atherosclerosis. It may be that epidemiological transition in this population is taking place rapidly with increase in prevalence of cardiovascular risk factors such as hypertension and diabetes mellitus. This is worrisome given the high burden of stoke in Nigeria [10,11] where the health system is inadequate to cope with its attendant challenges.

The frequency of moderate to severe atherosclerosis of 20.8% in ECCA in our study is lower than what was found in France, a developed nation, where it was almost 50%, although this finding was in patients with fatal stoke [12]. We found an overall prevalence of atherosclerosis of 73.3% which is also lower than what was recorded in a Danish population where the autopsied subjects had a rate of 94% [7]. The higher figure among Caucasians may reflect a greater frequency of atherosclerosis in Caucasians as compared to Nigerians.

A male predominance has been found in many studies [13,14] and our findings are consistent with these. The overall distribution of lesions in males was found to be 40.0% and this was significantly higher than in females who had an overall distribution of 33.3%. Premenopausal women are at low risk for atherosclerosis. By contrast, men and postmenopausal women are at increased risk [15]. The explanation is that hormonal factors such as oestrogen has been observed to play a significant role in slowing the progression of atherosclerotic lesions in females. However, differences in atherosclerosis between genders are inconsistent nor can it be easily explained.

The International Atherosclerosis Project conducted in1960-1964 found that the average extent of coronary and cerebral lesions was higher for men than for women in all the white populations; however, the gender difference was less striking or absent in the black populations [16,17].

In our study, increasing age is a strong risk factor for atherosclerosis, with a positive correlation between the two(r = 0.60; p < 0.001). Mild atherosclerosis (types I, II and III lesions) first appeared in the age group 20–24 years, while moderate atherosclerosis (type IV and V lesions) first appeared in the age group 25–29 years. Severe atherosclerosis (types VI, VII, VIII and IX lesions) was first noted in the age group 45–49 years. The most severe lesion occurred in the age group 60–64 years. This is in line with other studies in Western populations which have shown increase in atherosclerosis severity with age in all arterial beds with acceleration in severity from the fourth to the fifth decade of life [7,18]. Within the first two decades of life, atherosclerotic lesions are expected to be foam cell lesions and fatty streaks involving lesion growth by LDL-C accumulation which makes up types I-III lesions. Types I and II lesions, sometimes combined under the term early lesions, generally are the only ones that occur in infants and children, although they also occur in adults. Type III lesions may evolve soon after puberty and, in their composition, form the bridge between early and advanced lesions. By the third decade, lesions may have progressed to atheroma, which makes up type IV lesion [18].

Whether or not carotid artery atherosclerosis correlates with height is still unclear. We found no correlation between carotid lesions and height(r = 0.25; p > 0.05) although other workers have found a negative correlation with coronary atherosclerotic lesions [19,20]. Taller people with better lung function are less likely to develop atherosclerotic lesions and they have more favourable cardiovascular risk factor profiles [20,21]. Waist circumference and maximal AWT which are indexes for abdominal obesity, are known to increase the risk of developing atherosclerotic lesion [6]. Our study also showed a positive correlation of carotid atherosclerotic lesions with WC (r =0.60; p < 0.001) but not with AWT (r = 0.19; p > 0.05).

We found that the most frequent site for atherosclerosis was the right and the left carotid bifurcations followed by the right and the left internal carotid arteries. This distribution is similar to observations in a previous study [22]. The tendency for atherosclerotic plaques to occur at the carotid bifurcation is related to a number of factors, including geometry, velocity profile, and shear stress. It has also been demonstrated that plaque formation in the carotid artery bifurcation is increased in areas of low flow velocity, low shear stress and areas with high particle residence time, which are observed to facilitate particle such as LDL-C interaction with the arterial wall and decreased in areas of high flow velocity and elevated shear stress [7,23]. Post-mortem specimens have shown that atherosclerosis is particularly pronounced along the outer or lateral aspect of the proximal internal carotid artery and the carotid bulb [23,24]. This zone corresponds to areas of low velocity and low shear stress. Conversely, the medial or inner aspect of the cadaveric carotid bulb which was associated with high blood flow velocity and high shear stress in the flow model, were relatively free of plaque formation.

Foam cell lesions (types I and II lesions) are the most predominant lesions in carotid atherosclerosis [7]. The commonest lesions that we found were also mild (type I and type II) in 52.5% of the histological sections. The predominance of foam cell lesions in the common carotid artery is interesting because this artery is the preferred site for measurements of intima-media thickness (IMT) [25,26]. The reversibility of foam cell lesions may explain why the IMT can decrease in response to treatment with statins. A decrease in thickness would be difficult to explain if the thickening was fibrous, but if the bulk of the thickness was accounted for by foam cells, a decrease is much more likely. Foam cell lesions are not responsible for clinical events, but foam cell lesions are believed to be precursors of plaques in atherosclerosis-susceptible arterial segments. Studies observed that in the third decade of life the carotid bifurcation is dominated by foam cell lesions (types I and II) which are gradually replaced by lipid core plaques (type IV and V) in the following decades [25].

## Conclusions

Compared with four decades ago there has been an apparent increase in severity and extent of cerebral atherosclerosis especially after the age of 45 years in autopsies from our centre. This change in the amount of cerebral atherosclerosis over time is possibly due to the epidemiologic transition. This may worsen the rise in stoke incidence within this community and as such, great effort should be made to follow-up and manage CVD risk factors within the community.

Limitation of the study: A low autopsy rate was observed and this may be compounded by ethical issues and the religious beliefs of some faithful [27]. Furthermore, data on major risk factors are not available due to the retrospective nature of the study.

## Abbreviations

AWT: Abdominal wall thickness; AHA: American heart association; CVD: Cardiovascular disease; ECCA: Extra cranial carotid artery; WC: Waist circumference.

Institution to which the work should be attributed: Department of Pathology, University College Hospital, College of Medicine, University of Ibadan, Nigeria.

## Competing interests

The authors declare that there are no competing interests.

## Authors’ contributions

OOO conceptualized the study. OOO, EEI, OGO and AEEU were involved in the study design. EEI and OGO collected the data. EEI, OOO, OGO and AEEU were involved in the analysis and interpretation of the data. All authors read and approved the final manuscript.

## Pre-publication history

The pre-publication history for this paper can be accessed here:

http://www.biomedcentral.com/1471-2261/12/106/prepub
